# Fatherhood and Smoking Problems in Indonesia: Exploration of Potential Protective Factors for Men Aged 18–49 Years from the United Nations Multi-Country Study on Men and Violence

**DOI:** 10.3390/ijerph17196965

**Published:** 2020-09-23

**Authors:** Nurul Kodriati, Elli Nur Hayati, Ailiana Santosa, Lisa Pursell

**Affiliations:** 1 School of Health Sciences, National University of Ireland, H91 TK33 Galway, Ireland; n.kodriati1@nuigalway.ie (N.K.); lisa.pursell@nuigalway.ie (L.P.); 2 Faculty of Psychology, Post Graduate Program, University of Ahmad Dahlan, Yogyakarta 55166, Indonesia; elli.hayati@psy.uad.ac.id; 3 Department of Public Health and Community Medicine, Institute of Medicine, Gothenburg University, 405 30 Goteborg, Sweden

**Keywords:** men, masculinity, smoking, fatherhood, protective factor

## Abstract

Background: Despite the sustained high prevalence of smoking among Indonesian adult men, little is known about possible protective factors in this group. This study examined the relationship between key characteristics of masculinity (e.g., fatherhood status, being the main breadwinner or sole provider for the family) and current smoking behaviours (smoking status and cigarettes smoked per day (CPD)) among Indonesian men aged 18–49 years. Methods: In total, 2540 Indonesian men aged 18–49 participated in the United Nations Multi-Country Study on Men and Violence, 2012. Fatherhood status was categorised into three groups: nonfathers, new fathers and more experienced fathers. The association between fatherhood status and current smoking, as well as fatherhood status and cigarettes smoked per day (CPD), was estimated by employing logistic and zero-inflated negative binomial regressions, respectively. Results: Socioeconomic factors were associated with smoking behaviour among Indonesian adult men. The odds of smoking among new fathers and more experienced fathers were 2.3 (95% CI: 1.09–4.79) and 1.5 times (95% CI: 1.08–2.17) higher compared with nonfathers, respectively. Men who had a shared income with their partner or received income from their parents smoked 13% (95% CI 0.79–0.95) and 11% fewer CPD (95% CI 0.79–0.99) compared with men who were the main breadwinner, respectively. Conclusions: In this study, fatherhood represents an aspect of traditionally masculine roles, offering a new perspective for looking at smoking problems in Indonesia. Other key aspects of traditional masculinity characteristics, the breadwinner role, occupation and sources of family income had significant associations with smoking status and CPD. Men smoked fewer CPD as fathers and when sharing the financial responsibility for their family equally with their spouse.

## 1. Introduction

Globally, smoking is more prevalent among men than women with the ratio of men–women smokers varying across regions and demographic groups [1,2,3]. In addition to the burden of active smoking, second-hand smoke exposure is also high among men, affecting 33% of men worldwide [4]. Generally, men’s smoking behaviours have mostly been discussed within populations as nongendered behaviour. This situation can be contextualised within the lack of discussion on men and masculinity in general [5,6]. However, studies on masculinity and smoking have recently gained more focus over the last decade [7]. This focus is of growing importance when countries such as South Korea, China and Indonesia [8,9] have a prevalence of up to 10 times more men smokers compared with women smokers. 

In 2015, Indonesia was ranked the third highest country in male smoking prevalence [10], but it is the only country in Southeast Asia that has neither signed nor ratified the Framework Convention on Tobacco Control [9]. Associations between smoking and masculinity are deeply rooted in Indonesian culture and can be traced back to the colonial influences of Dutch society in the seventeenth century [11]; these associations have been exacerbated by extensive advertisements that promote smoking as an accepted norm and as a way to enhance masculinity [12]. Even though masculinity is a concept comprising multiple entities that can change over a man’s life course (Connell [13]) and that interplays with other factors such as race or class [14], the nature of smoking advertising still very much conveys an image of traditional and dominant masculine characteristics, such as being strong, tough and heroic [15]. These characteristics are very relevant to Indonesian men, especially among adolescent boys [16,17], and their use in advertising tends to undermine messages concerning the risks and harms of tobacco [18]. However, their attitudes towards smoking tend to be challenged when men start families and begin to identify as fathers [19,20,21].

Fatherhood status has been identified as one of the few protective factors against smoking [7]. Even though early fatherhood can be an extremely difficult and confusing situation for some men, their identity as fathers starts to develop after their first baby is born and progresses as they become more involved with their children’s daily care [22]. New fatherhood brings new responsibilities, with a major commitment and an urgency to obtain the necessary skills and knowledge to provide care for the infant and to support their partner in feeling confident and competent about her role as a mother [23]. The skills required in daily caring for their infant appear to be a crucial step in building confidence and satisfaction and are essential for fathers to spend more time with their babies [24]. It is this time spent with their children that has been identified as providing fathers with the time to reflect and identify their priorities, to consider greater self-care and engage in less risk taking [24], for example, not smoking around their children because it may expose them to passive smoking [25,26]. 

However, the socially constructed roles for fathers mainly orient them in a gendered division of labour, moving men towards being “a good breadwinner” [20,22,27,28]. This phenomenon is of no less relevance in Indonesia [16], where the concept of becoming a father only recently gained momentum, which can be illustrated by the relatively recently introduced celebration of Father’s Day in 2014 [29]. However, with the introduction since 2003 of only two days of paid paternal leave [30], there remains quite limited time for men to bond with their children. In these contexts, some men conflate their masculinity with workplace status or success, and different types of occupations become different markers of masculinities [5]. These socially prescribed roles often take men away from daily involvement with their children [28]. This social prescription is particularly valid when compared with the more recently emerged nurturant father roles, which have a much higher level of active involvement for men in their children’s daily lives [21,22,27]. Thus, from an equity perspective, Indonesian men have fewer opportunities to spend time with their children and develop their nurturing characteristics, which are important factors that may contribute to engaging in fewer risk-taking behaviours, including smoking.

Despite the sustained high prevalence of smoking among Indonesian adult men, little is known about the possible protective factors in this group. The aim of the current study was to explore the relationships between Indonesian men’s smoking behaviours and traditional aspects of masculinity, including fatherhood status, occupation and whether men were the main breadwinner or sole provider for the family, along with other socio-demographic characteristics, using a population-based survey.

## 2. Materials and Methods 

### 2.1. Study Design and Population

The present study used data from the United Nations Multi-Country Study on Men and Violence. Detailed information on the methodology can be found elsewhere [31,32]. The survey employed a multistage sampling strategy. The first stage was selecting the study sites. For political and practical considerations, Jakarta, Purworejo and Jayapura were chosen as the sites of the study. The second stage involved the selection of enumeration areas (i.e., the primary sampling units or clusters). Clusters were then divided into the three regions and then sampled with probability proportionate to size within each region. The number of clusters from each region was designed to reflect, as far as possible, the proportion of the population in that region, resulting in a representative sample of the respective survey populations. Thus, the design of this study provided a self-weighted sample for each site. Following this stage, households were randomly selected, followed by a chosen eligible individual randomly within each. In total, 3000 men were invited to participate in the survey, and a final response rate of over 85% was attained from three cities (Jakarta, Purworejo and Jayapura). Jakarta is the capital city of Indonesia and is representative of an urban area, while Purworejo represents a more rural area. Both of these cities are located on Java Island. Jayapura is on Papua Island and was chosen because this is where UN Women and UNFPA work due to the high prevalence of violence against women in this area.

The original questionnaire consisted of eight sections of sociodemographic characteristics and employment; childhood experiences; attitudes about relations between men and women; intimate relationships; fatherhood; health and well-being; policies; and sensitive and private questions (ranging from sexual relationships to criminal acts). Questions related to smoking were added in the Indonesia study and comprised smoking status, type of tobacco product used and number of cigarettes smoked per day. In total, 2561 cases were included in the current analysis. 

### 2.2. Measures

In the current study, the dependent variables were current smoking and number of cigarettes smoked per day (CPD), and the independent variables were fatherhood status, source of family income and sociodemographic variables (age, education, occupation and partnership status). Current smokers were men who smoked any tobacco products and had smoked in the past 30 days. CPD were derived from a question of how many average cigarettes the individual smoked per day. 

Fatherhood status had three categories: nonfathers, new fathers and more experienced fathers. Nonfathers were men who did not have any child. New fathers were defined as men who have one child under one year old. More experienced fathers were defined as men who have a child older than a year or more than one child. 

Other sociodemographic associated with smoking behaviours were included as covariates [33]. Age was categorised into three groups: 18–24 years, 25–34 years and 35–49 years. Education was based on the individual’s highest educational level attained and grouped into three categories: low (no schooling up to primary school), medium (incomplete and complete high school) and high (tertiary education). Partnership status was categorised into single/unmarried, married/cohabiting and divorced/widowed. Current occupations were grouped into three categories: nonmanual, manual and never worked/students. The main sources of family income were categorised as husband, spouse, both equally, parents and other.

### 2.3. Statistical Analysis

All statistical procedures employed STATA IC13.1 (Statacorp LLC Texas, TX, USA) and considered stratification by site and enumeration areas. The descriptive characteristics of the respondents are presented as the percentage of current smoking and mean CPD ± SD. Current smoking and CPD were stratified based on age, education, partnership status, occupational groups, fatherhood status and sources of family income. The differences among those groups were measured using chi-square and ANOVA tests for current smoking and CPD, respectively. 

Two regression analyses were performed. First, a logistic regression model was employed to examine the association between smoking status and fatherhood status, adjusting for other potential covariates. Bootstrap syntax was employed to overcome issues of potential bias. Secondly, since the distribution of CPD was over-dispersed due to an excessive number of zeros and unobserved heterogeneity, a zero-inflated negative binomial regression was performed to enable modelling of highly skewed data that contained a high percentage of zeros as the results of being non-smokers. In modelling CPD, all non-smokers were included in the model as zeros, providing a measure of how likely different groups were to smoke more or fewer cigarette across independent categories. The number of CPD would be zero-inflated by current smoking when men were non-current smokers. Odds ratios (OR) were reported for the logistic regression and rate ratios (RRs) for the count model (zero-inflated negative binomial regression), with 95% CIs for the analysis. 

## 3. Results

### 3.1. Characteristics Study Participants by Current Smoking and CPD

The overall prevalence of current smoking was 66.7%, with a mean CPD of 7.3 ± 7.8 (Table 1). Three characteristics (partnership status, type of occupations and fatherhood status) shared similar significant differences between current smoking and CPD. Three other characteristics (age, education and source of income) had different significance results between current smoking and CPD. 

In terms of CPD, the variance was greater than the mean, suggesting the presence of over-dispersion. The number of CPD tended to increase with age. On the other hand, current smoking also tended to be lower for those who had obtained a higher education. Single men, never worked/students and nonfathers tended to have the lowest prevalence of current smoking and fewer CPD compared with other categories of partnership status, occupational groups and fatherhood status, respectively. Interestingly, men who were the main breadwinner in the family had the highest mean number of CPD. 

### 3.2. Factors Associated with Current Smoking

Logistic regression was used to estimate the association between fatherhood status, sociodemographic factors and current smoking (Table 2). Findings shows that new fathers and more experienced fathers had 2.3- and 1.5-times greater odds of being a current smoker compared with men who were not fathers, respectively. Men aged 35–49 years had 36% lower odds of current smoking (95% CI 0.46–0.88) compared with those aged 18–24 years. Compared with those with a lower level of education, men with a medium level of education had 37% lower odds of current smoking (95% CI 0.52–0.76) and those with a high level of education had 64% lower odds of current smoking (95% CI 0.27–0.49). Current smokers who worked in the nonmanual and manual occupation groups had 1.7 (95% CI 21–2.33) and 2.4 times higher odds (95% CI 1.70–3.34), respectively, than men who never worked or were students. 

### 3.3. Factors Associated with CPD

The number of CPD was modelled using a negative binomial distribution; the outcomes are presented in Table 2. Fatherhood was not significantly associated with CPD. Men with a medium level of education smoked 1.1 times more CPD (95% CI 1.01–1.15) than men with a low level of education. Those who work in non-manual occupations smoked 1.2 times more CPD (95% CI 1.03–1.36). According to partnership status, divorced or widowed men smoked 1.5 times more CPD (95% CI 1.22–1.87) than single men. Finally, compared with men who were the main breadwinner, men who had a shared income with their spouse or received income from their parents smoked 13% (95% CI 0.79–0.95) and 11% fewer CPD (95% CI 0.79–0.99), respectively. For the zero-inflated group, the odds of CPD being in the certain zero group of smokers decreased significantly by −29.8 (95% CI −30.3; −29.3).

### 3.4. CPD by Different Fatherhood Status and Sources of Family Income

**Figure 1:**
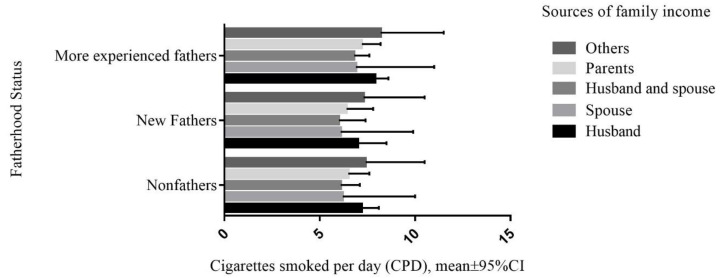
CPD and fatherhood status by sources of family income among nonfathers, new fathers and more experienced fathers.

In light of the source of family income, Figure 1 displays the mean CPD based on fatherhood status using sources of family income. Men who had shared income with their spouse tended to have the lowest mean CPD compared with men who reported other sources of family income. 

## 4. Discussion

The current study examined the relationship between key characteristics of masculinity (e.g., fatherhood status, being the main breadwinner or sole provider for the family) and current smoking behaviours (smoking status and CPD) among Indonesian men aged 18–49 years. The main findings showed dimensions of the breadwinner role, occupation and sources of family income had significant associations with smoking status and CPD. Manual and nonmanual workers were more likely to smoke and to smoke more per day than nonworkers or students; in addition, having equal sources of family income from both spouses was associated with smoking fewer CPD. For fatherhood status and partnership status, both new and more experienced fathers showed an increased likelihood of smoking, but this was higher for the more experienced than for new fathers. Being widowed or divorced was associated with increased smoking compared with being single.

In the context of traditional aspects of masculinity being used in tobacco advertising to promote and increase cigarette usage among men, these outcomes will be discussed in terms of their relevance for smoking prevention and cessation among Indonesian men, giving particular regard to the differences found between new fathers and more experienced fathers.

### 4.1. Smoking and Sociodemographic Factors 

The current study found younger Indonesian men and those with lower education levels had a greater likelihood of being a current smoker. This disparity in smoking behaviour among different socio-economic groups has been extensively identified by studies conducted in low- and middle-income countries [34,35], including Indonesia [33]. For example, older smokers are more likely to quit, and education is inversely associated with smoking in multiple settings [34]. A recent review on masculinity and smoking also supported the idea that men tend to moderate their smoking when they grow older in keeping with a changing vision that places less emphasis on the need to perform risky behaviours to appear masculine [7]. 

### 4.2. Smoking and New Fathers

In the current study, new fathers had a higher smoking prevalence than nonfathers. Fatherhood is a state spanning many years, starting from the time the first baby is born. The first year of fatherhood is a transitional phase for men who need to learn their new role and responsibilities as a father, during which protecting their babies from the harm of tobacco forms an important part [25]. Fatherhood might provide new fathers with an opportunity to reconsider their smoking behaviour after having a baby. Therefore, providing men with information on quitting smoking and facilitating cessation need further exploration to determine the most effective forms of intervention for men during this period.

Fatherhood is often measured quantitatively as the amount of time men spend with their children. According to Lamb, fathers’ quality of involvement with their children [22] is also important to offset men’s difficulty in achieving sufficient time and involvement with their children. Some men find it difficult to find activities to spend time with their baby and create the bonding needed [5]. This difficulty contrasts with women, who can continue after the child’s birth to maintain a bond through breastfeeding. 

However, the effect on smoking might be limited in a place where developing a fatherhood identity is not seen as a priority. Support for fathers during the period where men have their first child is an important time to help them go through this transition phase, for example, by providing paternal leave for them. In Indonesia, national laws recognise paid paternal leave for two days only [36]. In the current study, the findings showed that even though the majority—80%—took that paternity leave scheme, this length of time was considered to be very limited for fathers to develop their fatherhood identity and obtain the necessary skills to take care of their baby. 

### 4.3. Smoking and More Experienced Fathers

Fatherhood spans well beyond the infancy period; therefore, the present study also examined the relationship between smoking behaviour and being a more experienced father who has older children.

Compared to early fatherhood, more experienced fathers typically have a more active role, such as becoming their child’s playmate, guide or friend [37]. The time spent with their children and this active role with their children were argued to give men the time for reprioritising their activities and behaviours and, most importantly, to develop their fatherhood identity to become not only a provider but also a protector from any harm, including harm from tobacco.

Several other studies have supported the idea of fathers’ roles in interacting with their children in play, while mothers interact more in their daily care [22,37,38]. These fathers have more clarity and opportunities on how to be involved with their older children’s daily lives; they can have more responsibility by giving their children advice, discipline, spiritual guidance or examples of how to behave like a man for their boys and what kind of man their girls should choose later in life [27,37]. Therefore, the current study found that the increase in the likelihood of current smoking among more experienced fathers was not as high as for new fathers.

In this study, new or experienced fathers tend to be older than nonfathers. This was supported by our data with 61.5% of the nonfathers aged 18–24, 67.9% of the new fathers aged 25–34 and 63.4% of the experienced fathers aged 35–49. However, our results found that the odds of smoking among new or more experienced fathers were higher compared to nonfathers, and men aged 35–49 years showed lower odds of current smoking compared with men aged 18–24 years. One possible reason is that the average marriage age of men in Indonesia is around 22–24 years old. Thus, many experienced fathers could be aged 18–34 years old with lower odds of current smoking. In fact, smoking is less prevalent among older Indonesian men. As suggested by the results, the odds of smoking among fathers in Indonesia may differ by age group.

### 4.4. Smoking, Fatherhood and Breadwinner

Men often conflate their fatherhood role as with that of being the breadwinner [5], a concept that is strengthened by the traditional prescribed role where a father must be a good breadwinner [39,40]. Therefore, fathers tend to prioritise their work over their families and children. In the current study, most of the participants were the main contributors to family income, at almost 70%, indicating that the primary role they played was to be the breadwinner for their family. A recent study on contemporary masculinities in Indonesia supported that young Indonesian men’s constructions of masculinity oriented them to become a good provider for their family [16]. This role is often contradictory to that of a nurturant father because some men have difficulty finding time away from paid employment and often feel left out because they then have fewer opportunities to interact with their babies and children [37]. Thus, the two roles of being a breadwinner and a nurturing father often are at odds with each other for men; they experience conflicting obligations between paid work and family life [21]. Some authors have concluded that fatherhood/parenting presents an opportunity to try to quit or at least have the intention to quit [41]. Fatherhood also provides the opportunity to increase the receptiveness to smoking cessation among adult men [42]. Our results point to the need for further examination of the exclusion of men in parenting as a missed opportunity to address smoking problems among men.

The current study also found that men who share the breadwinner role with their wives tended to have a lower CPD compared with the other sources of family income. This suggests that these men who shared their financial responsibility with their spouse might have unique family characteristics, related to equally shared parenting, compared with families with different sources of income. When smoking is used as a means of coping with stress across a man’s life course [18,43,44], shared financial responsibility means lower stress for men. Thus, men could possibly reduce their CPD as they experience lower stress related to their family financial issues. Further exploration is necessary to gain more clarity on why it might provide benefits for reducing smoking among men. 

### 4.5. Public Health Implications and Recommendations

Along with other studies [19,43,45], it might be the case that smoking cessation interventions aimed at new and experienced fathers may help them reduce the amount they smoke and eventually quit because men who were fathers tended to smoke less than men who did not have children in this study. Future studies focusing on fatherhood and smoking could include other intermediary outcomes, such as attempts to quit smoking, changes in where the cigarettes are smoked or different smoking patterns to prevent children from being exposed to second-hand smoking, because these outcomes could be more realistic and relevant in evaluating the impact of fatherhood on smoking among men [19,43]. 

Further exploration of the role of fatherhood and smoking control among men could be beneficial if integrated within the implementation of a program of male involvement in childcare. An existing campaign by Suami SIAGA (alert husband) was introduced in 1998 to advocate for shared responsibility in birth preparedness [46]. The term “SIAGA” stands for “SIap” (ready/be prepared), “Antar” (take, transport) and “jaGA” (guard), which promotes men in supporting their wives in planning and attending the pregnancy, childbirth and after-birth visits. Additional information on the harms smoking poses to their wives and children could be disseminated during antenatal care visits and new-born check-ups.

### 4.6. Strengths and Limitations of the Study

The data used in the current study were sourced from a survey on men and violence, aiming to better understand men’s behaviour in an Indonesian setting. The results from the current study can contribute to the values of fatherhood status and its influence on smoking-related outcomes among men in Indonesia. Indonesia is one of the countries with a high smoking prevalence among men. To the best of our knowledge, the current study is the first one to explore the relationship between smoking, masculinity and fatherhood in Indonesia. Given the high prevalence of smoking and a high percentage of marriage among Indonesian men, the connections between masculinity and a father’s smoking behaviours might offer new perspectives for smoking control in Indonesia. 

The current study has three key limitations. Firstly, the survey was not designed specifically for studying smoking and fatherhood. Therefore, information on the signs of smoking dependence (always smoke tobacco or feel like smoking tobacco first thing in the morning) and that of the fathers’, friends’ or social networks’ smoking status could not be obtained. Moreover, the specific questions of the fathers’ involvement with their children was only completed by 30% of respondents in the original survey. Therefore, the association between men’s involvement with their children and their smoking behaviour could not be explored. This meant that factors such as how often fathers play or engage in activities with their children, talk about personal matters with their children, help any of their children with their homework and any negative interactions with them, such as how often they punish their children, were lacking in the current study. Data on stress related to fatherhood’s roles during the infancy period of their child were also not available in the current study and, in general, stress is a factor associated with smoking [43]. Further investigations that include these factors when examining men’s smoking behaviours are needed for future research. Secondly, The data in this study remain valid because even though the data were collected in 2012, there were no significant changes in terms of the high smoking prevalence among men relatively compared to women [1,2] nor in the overall masculine culture in Indonesia [16]. To the best of the authors’ understanding, the data in this study were derived from one of the existing surveys that specifically explores masculinity, fatherhood and smoking in an Indonesian context. Thirdly, this study was conducted in the three chosen cities based on the convenience, political and practical considerations of the country. Therefore, it is not possible to assume generalizability beyond the study sites. This study was also conducted only among men aged 18–49. Thus, the results are only generalizable to men aged 18–49.

## 5. Conclusions

Fatherhood, breadwinner role, occupation and sources of family income are associated with smoking behaviour among Indonesian adult men. In this study, those factors were framed as part of the masculine roles, which offer a new perspective for exploring smoking problems in Indonesia. Thus, integrating smoking prevention programs with existing health and family planning programs by involving men in childcare will have benefits by reducing the burden of smoking in an Indonesian context. However, men are more likely to benefit from being fathers when they share their breadwinner role with their spouse. This suggests that interventions focusing on how to reduce financial stress among men are necessary.

## Figures and Tables

**Table 1:** Current smoking and cigarettes smoked per day (CPD) by the characteristics of the respondents.

Characteristics (*n* = 2540)	Current Smoking *n* (%) ^1^	CPD ^2^ Mean ± SD ^3^
Overall	1695 (66.7)	7.3 ± 7.8
Age group	*p*-value = 0.238	*p*-value = 0.002 *
18–24	424 (64.1)	6.5 ± 7.2
25–34	561 (68.2)	7.7 ± 7.8
35–49	710 (67.2)	7.6 ± 8.1
Education level	*p*-value < 0.001 *	*p*-value < 0.008 *
Low	769 (75.6)	7.7 ± 7.4
Medium	804 (63.3)	7.3 ± 7.9
High	122 (48.4)	6 ± 8.6
Job category	*p*-value < 0.001 *	*p*-value < 0.001 *
Never worked/student	160 (51.8)	4.8 ± 6.5
Nonmanual	675 (62.6)	7.5 ± 8.4
Manual	860 (74.6)	7.9 ± 7.4
Partnership status	*p*-value < 0.005 *	*p*-value < 0.001 *
Single	502 (62.4)	6.4 ± 7.1
Married/cohabitated	1163 (68.9)	7.7 ± 8.0
Divorced/widowed	30 (65.2)	10.9 ± 10.7
Main sources of income	*p*-value < 0.009 *	*p*-value < 0.001 *
Husband	1029 (68.4)	8.0 ± 8.2
Spouse	12 (70.6)	7.2 ± 9.3
Both equally	291 (69.4)	6.9 ± 7.09
Parents	345 (60.6)	5.9 ± 6.7
Others	18 (60.0)	7 ± 7.9

^1^ Chi-square; ^2^ CPD: Cigarettes smoked per day; ^3^ ANOVA test; * *p*-value < 0.05.

**Table 2:** Logistic regression of current smoking and zero-inflated negative binomial regression of CPD with independent variables.

Variables	OR of Current Smoking	RR of CPD
	(95% CI)	(95% CI)
Fatherhood (Ref. Nonfather)		
New fathers	2.28 * (1.09–4.79)	0.85 (0.70–1.02)
More experienced fathers	1.53 * (1.08–2.17)	1.01 (0.89–1.15)
Age group (Ref. 18–24 years)		
25–34 years	0.8 (0.61–1.08)	1.05 (0.95–1.17)
35–49 years	0.64 * (0.46–0.88)	1.02 (0.90–1.15)
Education level (Ref. low)		
Medium	0.63 * (0.52–0.76)	1.08 (1.01–1.15)
High	0.36 * (0.27–0.49)	1.14 (0.99–1.31)
Job category (Ref. Never worked/student)		
Nonmanual	1.68 * (1.21–2.33)	1.19 * (1.03–1.37)
Manual	2.38 * (1.70–3.34)	1.07 (0.93–1.23)
Partnership status (Ref. single)		
Married/cohabitated	0.83 (0.56–1.24)	1.00 (0.87–1.16)
Divorced/widowed	0.82 (0.40–1.67)	1.51* (1.21–1.87)
Main sources of income (Ref. husband)		
Spouse	1.05 (0.36–3.02)	0.93 (0.55–1.55)
Both equally	1.08 (0.85–1.37)	0.87 * (0.79–0.95)
Parents	0.96 (0.69–1.33)	0.89 * (0.79–0.99)
Others	0.73 (0.32–1.65)	0.97 (0.71–1.32)
Inflated Current smoking ^a^		
Current smoker		−29.8 * (−30.3; −29.3)

CPD: Cigarettes smoked per day; RR: Rate Ratio; OR: Odds ratio; * *p*-value < 0.05. ^a^ The output for inflated results refers to the log odds from the logistic model, predicting whether or not a man is a certain zero.
